# Vanillic acid attenuates H_2_O_2_-induced injury in H9c2 cells by regulating mitophagy *via* the PINK1/Parkin/Mfn2 signaling pathway

**DOI:** 10.3389/fphar.2022.976156

**Published:** 2022-09-07

**Authors:** Manxue Mei, Haoxiang Sun, Jiayu Xu, Yimeng Li, Guiling Chen, Qihua Yu, Changsheng Deng, Wei Zhu, Jianping Song

**Affiliations:** ^1^ Artemisinin Research Center, Guangzhou University of Chinese Medicine, Guangzhou, China; ^2^ School of Food Science and Engineering, South China University of Technology, Guangzhou, China; ^3^ The Second Clinical School of Medicine, Guangzhou University of Chinese Medicine, Guangzhou, China; ^4^ Guangdong Provincial Hospital of Traditional Chinese Medicine, Guangzhou, China

**Keywords:** vanillic acid, H9c2, oxidative stress, mitophagy, Pink1/parkin

## Abstract

Vanillic acid, a phenolic compound mainly obtained from the foot of *Picrorhiza scrophulariiflora* Pennell, has been demonstrated to possess a cardiovascular-protective effect in previous studies. However, there is lack of research on vanillic acid protecting cardiomyocytes from oxidative stress injury by mediating mitophagy. In the present study, oxidative stress injury in the H9c2 cell line was induced by H_2_O_2_. Our results confirmed that vanillic acid mitigated apoptosis and injury triggered by oxidative stress, evidenced by the decline in production of reactive oxygen species and malondialdehyde and level of lactate dehydrogenase and the increase of superoxide dismutase and glutathione. The use of vanillic acid could also improve the polarization of mitochondrial membrane potential and decrease the cellular calcium level. After treatment by vanillic acid, impaired autophagy flux and mitophagy were improved, and the length of mitochondria was restored. Vanillic acid increased the expression of PINK1, Parkin, Mfn2, and the ratio of LC3-II/LC3-I and decreased the expression of p62. But, under the intervention of mitophagy inhibitor 3-MA, vanillic acid could not change the expression of PINK1/Parkin/Mfn2 and downstream genes to affect cell autophagy, mitophagy, and mitochondrial function. Our findings suggested that vanillic acid activated mitophagy to improve mitochondrial function, in which the PINK1/Parkin/Mfn2 pathway could be the potential regulatory mechanism.

## Introduction

Cardiovascular diseases (CVDs) are prevalent conditions around the world, and nearly 17.9 million people die from them every year according to the World Health Organization (WHO) (W.H.O, 2022). Oxidative stress (OxS) is involved in the pathophysiology of several CVDs, like myocardial ischemia-reperfusion injury, diabetic cardiomyopathy, arrhythmia, and heart failure (Bugger and Pfeil, 2020; Dhalla et al., 2020). The accumulation of reactive oxygen species (ROS) and the disorder of endogenous antioxidant defense are considered to be the cause of OxS. When OxS occurs in the heart, it may lead to some irreversible changes in DNAs, lipids, and proteins (Matsuda and Shimomura, 2013). In damaged mitochondria induced by OxS, malfunction of the respiratory chain will finally lead to less adenosine triphosphate (ATP) synthesis and the decline of integrity and quality in mitochondria, which contribute to apoptosis of cardiomyocytes (Chistiakov et al., 2018). So, increasing the quality of mitochondria in cardiomyocytes and decreasing the production of ROS are important to the treatment of CVDs. Also, autophagy is a mechanism that could effectively maintain the quality of organelles like mitochondria.

Autophagy, a conserved homeostatic mechanism which depends on the lysosome, plays a major role in the quality control of organelles (Kroemer, 2015). Mitophagy, a selective autophagic degradation of mitochondria mediated by the putative kinase 1 (PINK1)/Parkin pathway is closely related to CVDs (Ashrafi and Schwarz, 2013). Mitofusin 2 (Mfn2), the receptor of Parkin in damaged mitochondria, plays an important role in mitophagy downstream of Parkin (Gegg et al., 2010). Studies show that lack of mitophagy in Parkin-deficient mice (Parkin^−/−^) makes them more vulnerable to myocardial infarction. Mild OxS may trigger mitophagy to reduce the accumulation of ROS, but excessive OxS could inhibit mitophagy and finally trigger apoptosis of cells (Frank et al., 2012; Vegh et al., 2019). According to previous research studies, activating Parkin-dependent mitophagy could reduce injuries led by OxS and protect cells from different stimuli on cardiomyocytes, like CoCl_2_ and hypoxia/reoxygenation (H/R) (Sun et al., 2016; Cao et al., 2020). At present, research studies have confirmed that various compounds possess cardioprotective effects *via* regulating mitophagy (Xu et al., 2019; Hu et al., 2020; Luo et al., 2020).

**FIGURE 1:**
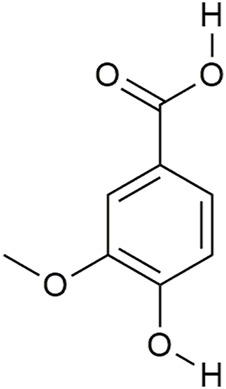
Structure of vanillic acid.

Vanillic acid (4-hydroxy-3-methoxybenzoicacid (VA) Figure 1), a cinnamic acid derivative, exists widely in nature and is found abundantly in several Chinese herbal medicines like *Picrorhiza scrophulariiflora* Pennell, *Atractylodes macrocephala*, and *Coptis chinensis*. VA has a strong antioxidant effect and demonstrates therapeutic potential against CVDs, which could reduce the production of intracellular ROS and reduce myocardial cell damage caused by hypoxia and reoxygenation (Luo et al., 2020; Kaur et al., 2022). At present, research about VA effects on cardioprotective focuses on reducing apoptotic rate and lipid peroxidation (Stanely Mainzen Prince et al., 2011), but there is lack of research studies on effects for mediating mitophagy.

Therefore, this study explored the cardioprotective effect against OxS of VA by regulating mitophagy *via* the PINK-1/Parkin/Mfn2 pathway.

## Materials and method

### Cell culture and treatment

The rat cardiomyocytic H9c2 cell line was obtained as a gift from Dr. Banhan Ding from the Second Clinical College of Guangzhou University of Chinese Medicine. Cells were cultured in Dulbecco’s modified Eagle’s medium (DMEM, Gibco Life Technologies, Lofer, Austria) with 10% fetal bovine serum (FBS) and 1% penicillin/streptomycin. Also, cells were maintained at 37°C in a incubator with an atmosphere of 5% CO_2_. Oxidative stress in H9c2 cells was induced by 600 μM hydrogen peroxide (H_2_O_2_) for 3 h in the same culture conditions mentioned above. Vanillic acid was purchased from Yuanye Biotechnology Co. Ltd., China.

### Cell viability assay and cytotoxicity assay

After being stimulated by 600 μM of H_2_O_2_, cell viability inhibition was determined by the MTT (methylthiazolyldiphenyl-tetrazolium bromide, MTT, Sigma (United States) assay. MTT was dissolved in 0.8% NaCl solution to a concentration of 5 mg/ml. Lactate dehydrogenase (LDH) activity was measured to estimate the cytotoxicity with an LDH Cytotoxicity Kit (Beyotime, China).

### Assessment of apoptosis

Apoptosis of H9c2 cells was determined by using an Annexin V-FITC Apoptosis Detection Kit, performed according to the manufacturer’s instructions (Beyotime, China). Cells were digested by trypsin and collected in 1.5 ml Eps and centrifuged at 1,000 rpm for 5 minutes. Then, the cells were incubated in 5 μL Annexin V-FITC and 10 μL PI solution for 20 minutes at room temperature. The stained cells were analyzed by flow cytometry.

### Determination of lactate dehydrogenase and intracellular Ca2+

Lactate dehydrogenase (LDH) activity was measured to estimate the cytotoxicity with a LDH Cytotoxicity Kit (Beyotime, China). The level of intracellular Ca2+ was determined by flow cytometry after being incubated with fluorescent Fluo-4/AM according to the recommendations from the manufacturer (Invitrogen, United States).

### Determination of reactive oxygen species, malonaldehyde, and superoxide dismutase

Cells were collected as per the protocol listed in 2.3. ROS was assessed by flow cytometry with an ROS assay kit (Sigma, United States). Intracellular MDA and SOD were determined by assay kits (Beyotime, China). After cells were analyzed according to the instructions from the manufacturers, concentrations of MDA and SOD were examined by sample absorbance using a spectrophotometer at 520 nm.

### Mitochondrial oxygen consumption

Oxygen consumption rates (OCRs) were measured with a Seahorse XF24 Extracellular Flux Analyzer (Agilent Technologies, United States). H9c2 cells were seeded in a Seahorse 24-well at a density of 10,000 per well. Before the day of the experiment, the probe plate was hydrated with a calibration liquid provided by Agilent. On the day of the experiment, the culture medium was removed after cell treatment and then each well was washed twice with Seahorse XF basic culture medium and incubated in a CO_2_-free incubator for 30 minutes. After that, oligomycin, antimycin A/rotenone, and FCCP were successively added to the probe plate for OCR detection.

### Measurement of mitochondrial membrane potential

Mitochondrial membrane potential (MMP, ΔΨM) of H9c2 cells was measured by flow cytometry and an inverted fluorescence microscope (Canon, Japan) using a mitochondrial membrane potential assay kit (Beyotime, China).

### GFP-mRFP-LC3 adenovirus infection in H9c2

Fluorescent GFP-mRFP-LC3 adenovirus (MOI = 100) was transduced into H9c2 cells for 8 hours to measure the autophagy flux following the instructions of the manufacturer (Hanbio, China). After the adherent cell growth, the H9c2 cells were cultured with a complete medium added with 2 μg/ml puromycin. The autophagy flux was determined by using a confocal microscope (Zeiss, Germany).

### Determination of the shape of mitochondrion and mitophagosome formation

To detect the length and number of mitochondria in cardiomyocytes, H9c2 cells were incubated with Mito Tracker Green (MTG, Beyotime, China) for 20 min at 37°C in darkness, followed by confocal imaging of mitochondria. MTG and Lyso Tracker Red (LTR, Beyotime, China) were used to stain H9c2 cells together, and the colocalization of these two fluorescent dyes represents the formation of mitophagosomes. After staining, cells were observed by using a confocal microscope, and the merged images were used for further analysis. Fluorescent images were analyzed with Fiji/ImageJ software (National Institutes of Health, United States).

### Western blot analysis

H9c2 cells were homogenized in the pre-cooled RIPA lysate containing 1% protease inhibitor (Roche, Germany) and 2% protein phosphatase inhibitor cocktail (Roche, Germany). Samples were centrifugated at 13,500 rpm for 15 min at 4°C. The concentration of protein in supernatants after centrifugation was determined by a BCA assay kit (Thermofisher, United States). 40 mg protein of each sample was separated on 10% or 12% sodium dodecyl sulfate polyacrylamide gel electrophoresis (SDS-PAGE) gels and then transferred onto a polyvinylidene fluoride (PVDF) membrane. After being blocked in 2% skim milk (Cell signaling technology), the PVDF membranes were incubated with antibodies at 4°C overnight. The antibodies incubated with PVDF membranes were as follow: PINK1 (D8G3) Rabbit mAb (Cat: 6946S, Cell Signaling Technology, United States), Parkin (Prk8) Mouse mAb (Cat:4211, Cell Signaling Technology, United States), SQSTM1/p62 Antibody (Cat:5114, Cell Signaling Technology, United States), LC3A/B Antibody (Cat:4108, Cell Signaling Technology, United States), Mitofusin-2 (D2D10) Rabbit mAb (Cat:9482, Cell Signaling Technology, United States), Bcl-2 mAb (Cat:T40056, Abmart, China), Bax Antibody (Cat:2772, Cell Signaling Technology, United States), Caspase-3 Antibody (Cat:9662, Cell Signaling Technology, United States), and β-Actin (13E5) Rabbit mAb (Cat:4970, Cell Signaling Technology, United States). Chemical luminescence was used to detect protein bands. Quantification of immunoblots was performed using Image Lab software (Biorad, CA).

### Data and statistical analysis

All results are shown as means ± SEM. Analysis of statistical data was conducted with GraphPad Prism 9.0 software (GraphPad Software). ANOVA with Dunnett’s post hoc test was used for statistical analysis. Statistical significance was defined as *p* < 0.05.

## Results

### VA attenuated H_2_O_2_-induced injury in H9c2 cells

**FIGURE 2:**
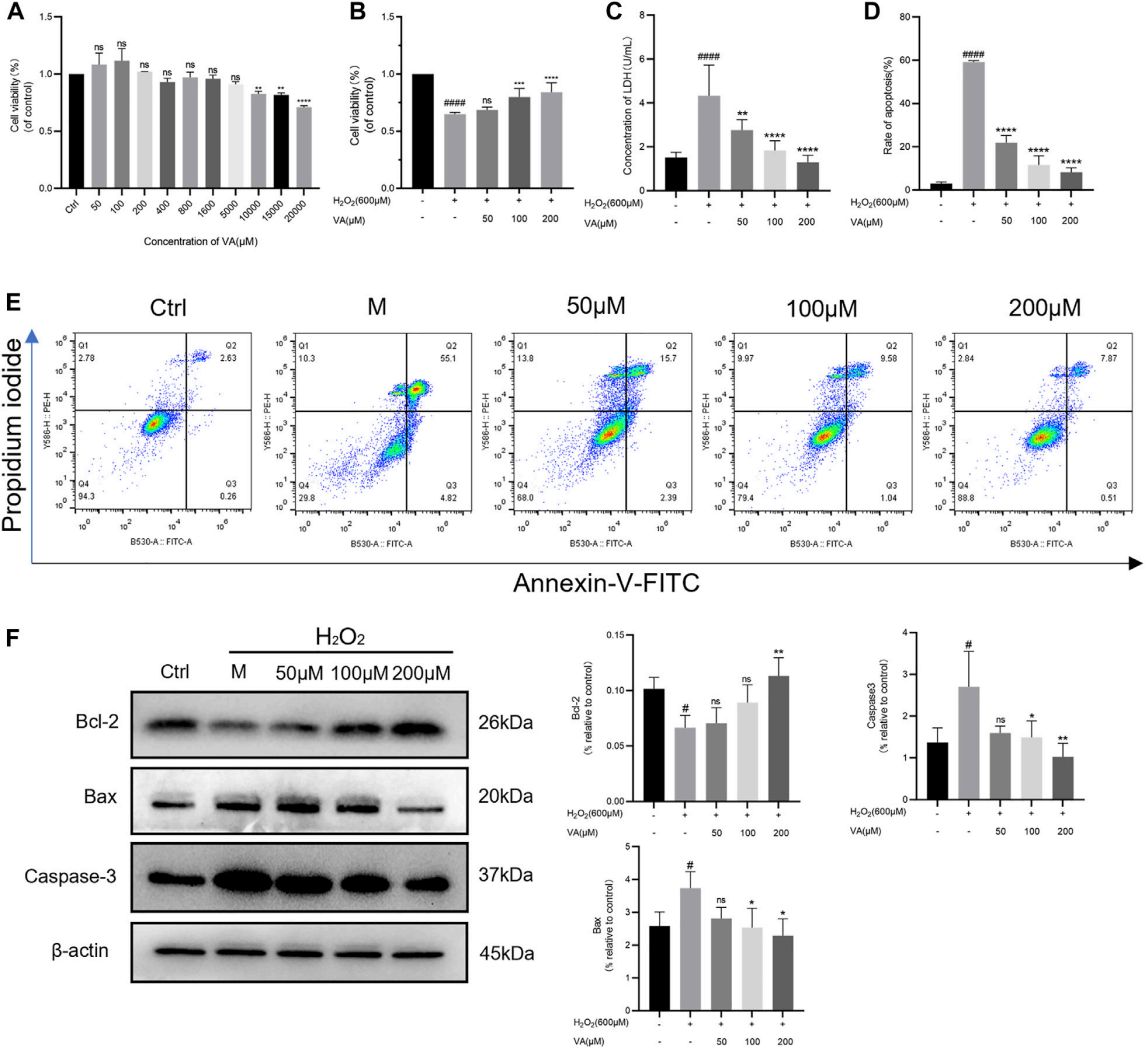
VA attenuated H_2_O_2_-induced injury in H9c2 cells. **(A)**VA was safe up to a concentration of 10,000 μM. **(B)** 50–200 μM VA increased cell viability. **(C)** VA decreased the level of LDH. **(D,E)** VA decreased apoptotic rate tested by flow cytometry. **(F)** The bands of Bcl-2, Bax and Caspase-3 and the expression of them were detected using western blotting. All data were displayed as mean ± SEM, n = 3. ^####^ *p* < 0.0001 *versus* the control group; *p < 0.05, **p < 0.01, ***p < 0.001, and ****p < 0.0001 *versus* the M group.

VA was found to have no effects on the viability of H9c2 cells in concentrations up to 10,000 μM. The H9c2 cells were treated with H_2_O_2_ for 3 hours. The results of MTT indicated that treatment of H_2_O_2_ dramatically reduced cell viability (*p* < 0.0001, *versus* the control, Figure 2). VA (50–200 μM) had a dose-dependent protective effect on H9c2 stimulated by H_2_O_2_ and the release of LDH. Hence, we chose a concentration of 50, 100, and 200 μM in the following examinations.

Disruption of the redox balance of the cardiomyocytes would result in the activation of apoptotic pathways. So, flow cytometry was used to detect the effects of VA on apoptosis of the H_2_O_2_ injured H9c2 cell line. After being incubated with H_2_O_2_, the rate of apoptosis was significantly increased, which could be attenuated by VA dependent on dose (Figures 2D and E). To further define the implication of VA on apoptosis, caspase-3, Bcl-2, and Bax were analyzed by Western blot. As the results showed that the expression of caspase-3 and Bax increased and Bcl-2 decreased after H_2_O_2_ treatment, intervention of VA reversed this phenomenon (Figure 2F).

### Effects of VA on oxidative stress biomarkers

**FIGURE 3:**
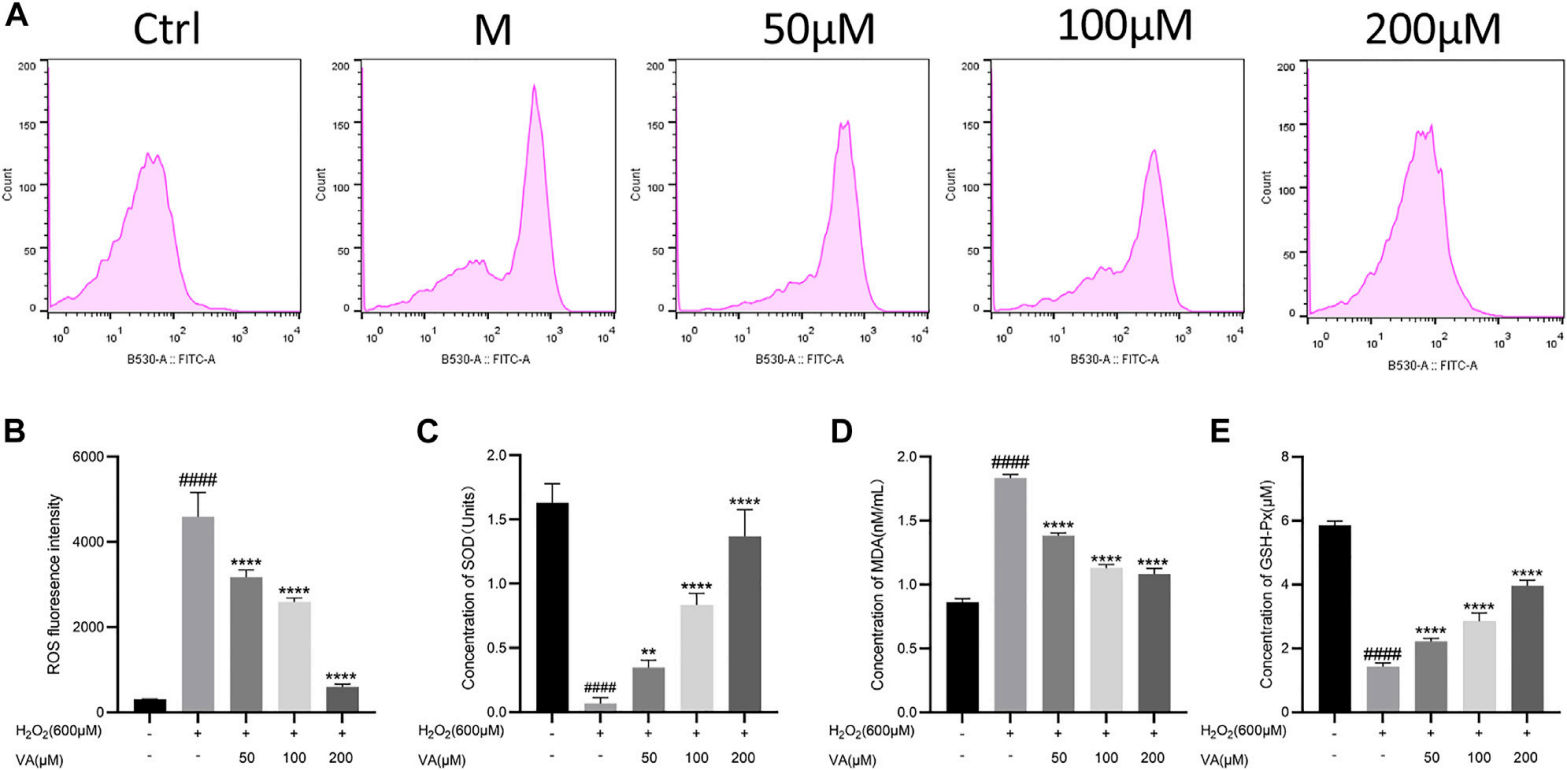
Effects of VA on the level of ROS, MDA, SOD, and GSH-Px in the H9c2 cells. **(A,B)** VA decreased the intensity of ROS fluorescence. **(C)** VA increased the concentration of SOD. **(D)**VA decreased the level of MDA. **(E)** VA increased the level of GSH-Px. All data were displayed as mean ± SEM, n = 3. ^####^ *p* < 0.0001 *versus* the control group; *p < 0.05, **p < 0.01, ***p < 0.001, and ****p < 0.0001 *versus* M group.

To explore VA effects on the redox system, the concentration of biomarkers of oxidative stress was further examined. A large amount of ROS accumulates in cells when oxidative stress exceeds the normal metabolic level of cells and finally aggravates the oxidative injury. MDA, the end-product of polyunsaturated fatty acid metabolism, could damage the mitochondrial respiratory chain. SOD and GSH-Px are the main antioxidant enzymes in cells, which could maintain the balance of oxidation. In this test, after treatment of VA, ROS generation and concentration of MDA were reduced in H9c2 cells compared to cells in the M group (Figures 3A, B, and D). The decreased levels of SOD and GSH-Px induced by H_2_O_2_ were reversed by VA compared to those in the M group.

### Effects of VA on the intensity of mitochondrial membrane potential and the level of intracellular free Ca^2+^ in H9c2 cells

**FIGURE 4:**
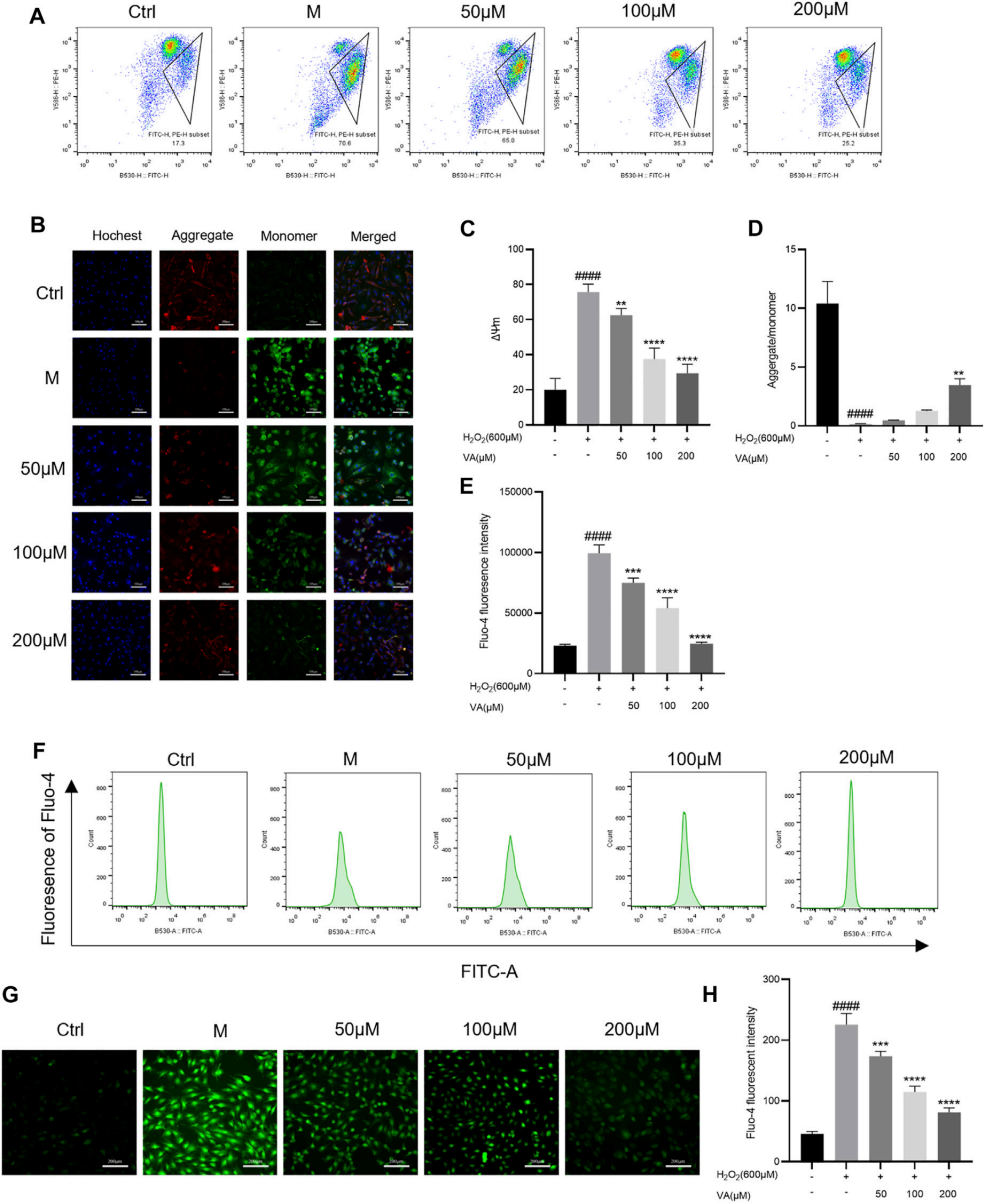
Effects of VA on the depolarization of MMP and the level of intracellular free Ca^2+^. **(A–D)** VA inhibits the depolarization of MMP after stimulating of H_2_O_2_. **(E–H)** VA treatment decreased the level of intracellular free Ca^2+^. All data were displayed as mean ± SEM, n = 3. ^####^ *p* < 0.0001 *versus* the control group; *p < 0.05, **p < 0.01, ***p < 0.001, and ****p < 0.0001 *versus* M group.

Oxidative stress is closely linked with disruptions in MMP and level of Ca ^2+^ in H9c2 cells. Damage of the mitochondria led by oxidation would cause the decrease of MMP. So, we tested the alternation of MMP and intracellular free Ca^2+^ after being incubated with H_2_O_2_ by inverted fluorescence microscopy and flow cytometry. VA treatment attenuated the depolarization of MMP compared to the control group (Figures 4A–D). The level of intracellular free Ca^2+^ decreased after treatment with VA (Figures 4E–H).

### VA activates mitophagy to protect mitochondrial function against H_2_O_2_-induced injury in H9c2 cells

Mitophagy, belonging to selective autophagy, is instrumental in mitochondrial quality control. Defective autophagy and mitophagy may lead to accumulation of intracellular damage and finally malfunction of the mitochondria. Damaged mitochondria, which are shorter in length than normal ones, have a worse respiratory ability and fail to produce enough ATP for cell function (Sun et al., 2021). So, we tested the influence of H_2_O_2_ treatment on autophagy flux, mitophagy, and function of the mitochondria. To further confirm that VA could improve the quality of the mitochondria *via* regulating autophagy flux and mitophagy, we used 3-methyladenine (3-MA, a kind of inhibitor of mitophagy) for comparison.

**FIGURE 5:**
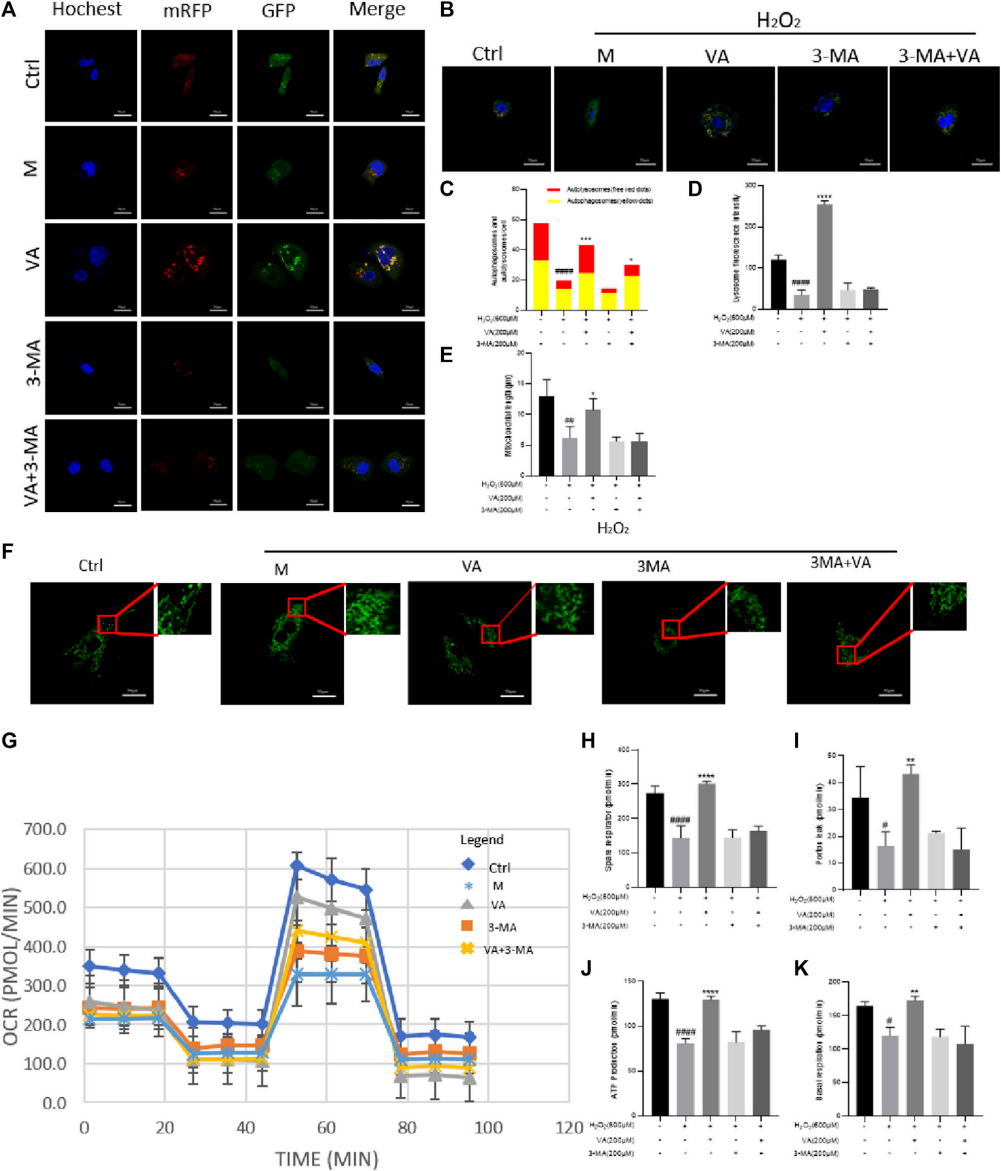
Effects of VA on autophagy, mitophagy, and OCRs. **(A,C)** H9c2 cells stably expressing mRFP-EGFP-LC3B were transfected with the vector. VA treatment increased the number of both red and yellow puncta and autophagy flux injured by H_2_O_2_. After treatment of 3-MA, autophagy flux was inhibited, and VA could not reverse this phenomenon. **(B,D)** Mitophagy was examined by Lyso Tracker (red) and Mito Tracker (green) staining together, and the fluorescence intensity was assessed by ImageJ. After being incubated with H_2_O_2_, the number of lysosomes reduced. VA treatment increased the quantity and fluorescence intensity of lysosomes, but it could not improve the reduction caused by treatment of both H_2_O_2_ and 3-MA. **(E,F)** Mito Tracker (green) was used to detect the mitochondrial length. H9c2 cells injured by H_2_O_2_ were shorter and rounder than normal cells, and VA could increase the number of mitochondria longer in length. **(G–K)** Agilent Seahorse kit was used to examine the mitochondrial basal respiration, residual respiration, proton leakage, and ATP. VA improved the mitochondrial respiratory function of oxidative stress injury, but the effect of VA on the mitochondrial respiratory function decreased significantly after adding 3-MA. All data were displayed as mean ± SEM, n = 3. ^####^ *p* < 0.0001 *versus* the control group; ***p < 0.001 and ****p < 0.0001 *versus* M group.

As a result, we found that H_2_O_2_ and 3-MA treatment could decrease the autophagy flux at an early state of autophagy, impair the mitophagy in H9C2 cells, and decrease their length compared to the control group (Figure 5).

### VA-activated mitophagy *via* the PINK1/parkin/mfn2 pathway

**FIGURE 6:**
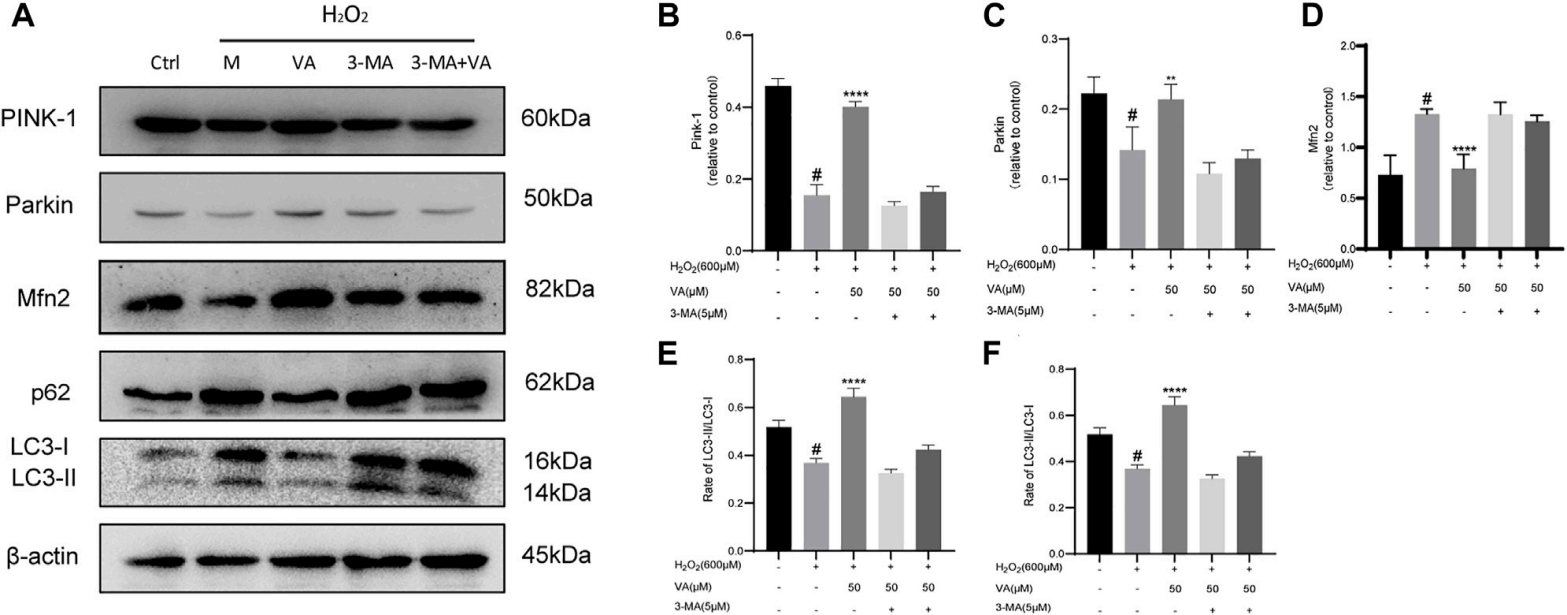
VA activates mitophagy through regulating PINK1/Parkin. **(A–F)** Expression of PINK1, Parkin, p62, LC3-II/LC3-1, and Mfn-2 was examined by Western blot. 3-MA was used as an inhibitor of mitophagy. All data were displayed as mean ± SEM, n = 3. ^####^ *p* < 0.0001 *versus* the control group; ***p < 0.001 and ****p < 0.0001 *versus* M group.

In order to determine that VA could affect mitophagy by dependence on PINK1/Parkin, we detected the expression of PINK1, Parkin, p62, LC3-II/LC3-I, and Mfn2. The results showed that 600 μM of H_2_O_2_ treatment reduced the expression of PINK-1, Parkin, and Mfn2 and the ratio of LC3-II and LC3-I and caused the accumulation of p62. Though use of VA improved this situation, VA could not have similar effects after the use of 3-MA. Thus, VA could activate autophagy and mitophagy *via* activating PINK-1 and Parkin pathways, reduced the accumulation of autophagy-related protein p62, increased the expression of Mfn-2, and increased the ratio of LC3-II/LC3-I (Figure 6).

## Discussion

In the present study, we demonstrated that mitophagy dependent on PINK1/Parkin/Mfn2 of H9c2 cells exposed to H_2_O_2_ was impaired, which contributed to higher oxidative stress and apoptosis. VA treatment could reverse these when induced by H_2_O_2_.

Mitochondria, organelles that undergo repeated fission and fusion to maintain their function, are the main location of cells for producing ATP *via* oxidative phosphorylation (OXPHOS) and controlling the local Ca^2+^ homeostasis to maintain normal cellular functions (Parra et al., 2011; Porporato et al., 2018; Shen et al., 2019). Rhythmic cardiac contractions are supported by high energy consumption, ultimately relying on mitochondrial function and morphology (Oka et al., 2011; Tahrir et al., 2019). Previous studies showed that the function of elongated mitochondria is more significant than that of rounded ones (Sun et al., 2021). As an oxidative metabolite, H_2_O_2_ production is well-correlated with the electron transport chain on the mitochondrial ridge and has a major impact on metabolism and function in cells as a messenger to deliver OS signals (Sies, 2017). Proton leakage refers to the mechanism of mitochondria returning directly through their inner membrane, which can directly affect the synthesis of mitochondria and can be used to evaluate mitochondrial function (Murphy, 1989). In the defense of reactive oxygen species, mitochondria increase the proton leakage of the mitochondrial inner membrane and reduce the transmembrane potential through uncoupling, stimulate the consumption of O_2_, and alleviate O^2^ ˉ(Yungang Zhao, 2001). In this study, 200 µM of H_2_O_2_ was applied to induce damage led by OS. Also, finally we found that after H_2_O_2_ treatment, the apoptosis rate increased with higher expression of Bax and caspase-3 and lower expression of Bcl-2. Meanwhile, severe OS resulted in the damage of MMP and overload of intracellular calcium.

Autophagy is a conserved evolutionary mechanism in cells responsible for balance generation and degeneration of organelles (Klionsky et al., 2016). Mitophagy, a typical selective autophagy targeting mitochondria, plays a vital role in maintaining mitochondrial function and quality (Galluzzi et al., 2017). Previous studies suggest that impaired autophagy and mitophagy could negatively influence the cardiac function of animals. Under the condition of nutritional stress, mitochondrial autophagy can alleviate the toxic effects of dysfunctional mitochondria on the heart, such as producing 10 times more ROS than normal mitochondria, which could directly attack mitochondrial proteins and DNA, aggravating mitochondrial dysfunction (Qiu et al., 2019; Yang et al., 2020). After administration of 3-methyladenine or non-enzyme a (both are inhibitors of mitochondrial mitophagy) to rats with myocardial infarction, the area of lesion sites increased, but the treatment of drugs with mitophagy activation, such as rapamycin or resveratrol, could reverse the myocardial injury in animals (Wu et al., 2014). In the present study, treatment of 600 μM of H_2_O_2_ inhibited mitophagy of H9c2 cells, reduced the autophagy flow, and caused the disorder of mitochondrial energy metabolism of cardiomyocytes. Therefore, regulating mitophagy represents a therapeutic target of OS injury.

**FIGURE 7:**
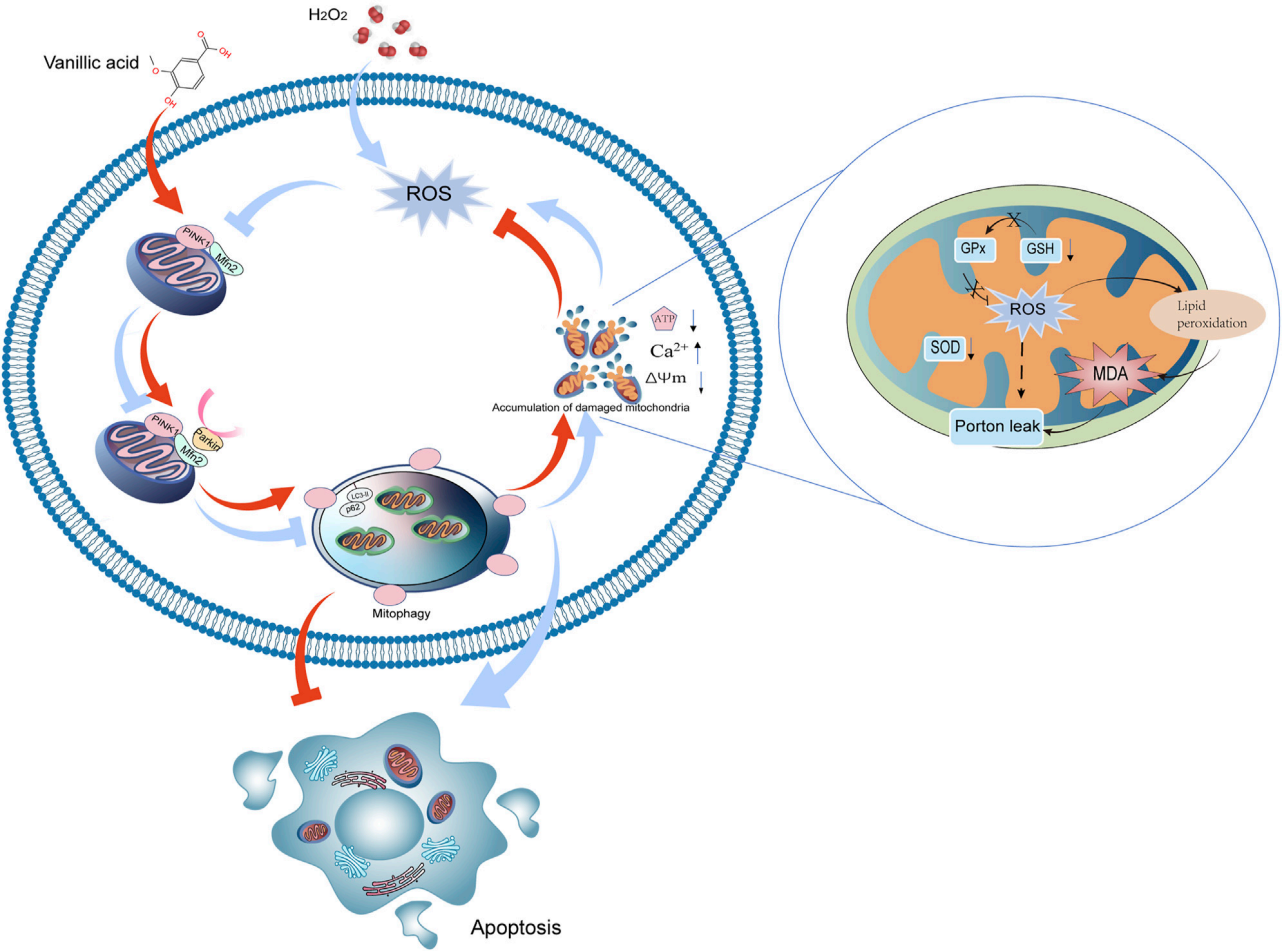
Mechanism of VA regulating mitophagy by the PINK1/Parkin/Mfn2 pathway.

Phosphatase and tensin homolog (PTEN)-induced kinase 1 (PINK1)/Parkin pathway is a classical mitophagy signal pathway (Song et al., 2018). PINK1 is a serine/threonine kinase, whose translocation to the inner mitochondrial membrane depends on the normal MMP (Eiyama and Okamoto, 2015). Parkin, a kind of E3 ubiquitin linkage encoded by the PARK2 gene, is widely expressed in the brain and heart, with a wide range of biological activities (Eiyama and Okamoto, 2015). Generally, Parkin is located in the cytoplasm, and its ubiquitin ligase activity is inhibited. After cell stress, PINK1 recruits Parkin to damaged mitochondria and phosphorylates it (P-Parkin). Substrate proteins on the mitochondrial membrane as mitochondrial fusion protein 2 (Mfn2) were ubiquitinated by P-Parkin (Rakovic et al., 2011). Mfn2-deficient mice suffer from respiratory dysfunction due to damaged mitochondrial accumulation (Chen and Dorn, 2013). Microtubule-associated protein 1 light chain 3 (LC3) aptamer protein recognizes ubiquitinated substrate protein, binds LC3 into autophagy, binds to lysosome, and finally degrades damaged mitochondria (Montava-Garriga and Ganley, 2020). Protein 62 (p62) is the substrate of autophagy, and impaired autophagy contributes to accumulation of p62 (Maroni et al., 2014). We found that exposure to H_2_O_2_ could reduce the expression of PINK1, Parkin, Mfn2 and the ratio of LC3-II/I and increased the expression of p62 (Figure 7).

Vanillic acid has an excellent antioxidant effect (Kaur et al., 2022). Previous studies have proven that VA could regulate adenosine 5‘-monophosphate (AMP)-activated protein kinase (AMPK- α2), nitric oxide synthase (eNOS), or endothelin 1 (ET1) to improve the oxidative stress injury of IR injury could also improve the symptoms in hypertensive rats (Kumar et al., 2014). Nevertheless, the protective and therapeutic effects of vanillic acid on cardiovascular disease still need further clarification.

## Conclusion

Overall, this study shows that VA could improve the function of mitochondria in H9c2 cells with oxidative injury *via* activating mitophagy through the PINK1/Parkin/Mfn-2 pathway, revealing the potential mechanism of VA’s protective effect on the heart. This study may help develop new applications of VA in the prevention and treatment of CVDs.

## Data Availability

The original contributions presented in the study are included in the article/Supplementary Material; further inquiries can be directed to the corresponding authors.

## Abbreviations

AMPK- α2, adenosine 5‘-monophosphate (AMP)-activated protein kinase; ATP, adenosine triphosphate; CVDs, cardiovascular diseases; ET1, endothelin 1; GSH, glutathione; H_2_O_2_, hydrogen peroxide; H/R, hypoxia/reoxygenation; LC3, light chain 3; LDH, lactate dehydrogenase; MDA, malondialdehyde; Mfn2, mitofusin 2; MMP, mitochondrial membrane potential; eNOS, nitric oxide synthase; OCRs, oxygen consumption rates; OXPHOS, oxidative phosphorylation; OxS, oxidative stress; PTEN, phosphatase and tensin homolog; PINK1, putative kinase 1; ROS, reactive oxygen species; SOD, superoxide dismutase; VA, vanillic acid; WHO, World Health Organization; 3-MA, 3-methyladenine.
